# Smart Chemical Sensor and Biosensor Networks for Healthcare 4.0

**DOI:** 10.3390/s23125754

**Published:** 2023-06-20

**Authors:** Lawrence He, Mark Eastburn, James Smirk, Hong Zhao

**Affiliations:** 1 Princeton High School, Princeton, NJ 08540, USA; 2 Gildart Haase School of Computer Sciences and Engineering, Fairleigh Dickinson University, Teaneck, NJ 07666, USA

**Keywords:** chemical sensors, biosensors, Industry 4.0, Healthcare 4.0, wearable devices, body chemical sensors and biosensor networks (BSNs), sensor, wearable data collector, tennis elbow, runner’s knee, shin splints, heel inflammation, blood flow velocity, successful number of readouts, monitoring time

## Abstract

Driven by technological advances from Industry 4.0, Healthcare 4.0 synthesizes medical sensors, artificial intelligence (AI), big data, the Internet of things (IoT), machine learning, and augmented reality (AR) to transform the healthcare sector. Healthcare 4.0 creates a smart health network by connecting patients, medical devices, hospitals, clinics, medical suppliers, and other healthcare-related components. Body chemical sensor and biosensor networks (BSNs) provide the necessary platform for Healthcare 4.0 to collect various medical data from patients. BSN is the foundation of Healthcare 4.0 in raw data detection and information collecting. This paper proposes a BSN architecture with chemical sensors and biosensors to detect and communicate physiological measurements of human bodies. These measurement data help healthcare professionals to monitor patient vital signs and other medical conditions. The collected data facilitates disease diagnosis and injury detection at an early stage. Our work further formulates the problem of sensor deployment in BSNs as a mathematical model. This model includes parameter and constraint sets to describe patient body characteristics, BSN sensor features, as well as biomedical readout requirements. The proposed model’s performance is evaluated by multiple sets of simulations on different parts of the human body. Simulations are designed to represent typical BSN applications in Healthcare 4.0. Simulation results demonstrate the impact of various biofactors and measurement time on sensor selections and readout performance.

## 1. Introduction

Industry 4.0 is the fourth industrial revolution. Its initial focus is on improving and optimizing manufacturing processes and supply chains. With its rapid development, Industry 4.0 has brought deep impacts to different aspects of society and our lives. In the healthcare sector, Industry 4.0 has promoted many advanced technologies to improve human health via advanced technologies [1,2,3]. This effort boosts a new concept, which is referred to as Healthcare 4.0.

Traditional healthcare practice relies heavily upon physical appointments, in-person visits, manual examinations, and treatment procedures with deep human involvement. Healthcare 4.0 takes advantages of many aspects from Industry 4.0 [4]. It synthesizes medical sensors, artificial intelligence (AI), big data, the Internet of things (IoT), machine learning, and augmented reality (AR) to transform healthcare with tech-driven applications and processes [5,6]. Healthcare 4.0 creates a smart health network by connecting patients, medical devices, hospitals, clinics, medical suppliers, and other healthcare-related components [7,8]. In this smart network, medical and care delivery decisions are made with much less human involvement; in some cases, even without any human participation [9,10].

As a key platform to collect healthcare information, body chemical sensors and biosensor networks (BSNs) [11,12,13] are essential to detect bio and medical data necessary for Healthcare 4.0. BSN systems rely on various types of chemical sensors and biosensors to sample, detect, and communicate physiological measurements of human bodies [14,15]. Chemical sensors are often utilized to detect the content and concentration of human body liquid such as PH value, Ca+ concentration, glucose concentration, etc. [16,17]. In the meantime, biosensors are widely used to detect antigens, antibodies, enzymes, hormones, DNA, RNA, as well as microbes [18,19,20]. With technological advances and collaborations to overcome the nontechnical barriers, BSNs are expected to demonstrate value for Healthcare 4.0 users to resolve impediments related to inconvenience, invasiveness, and general discomfort. The global BSN market is predicted to grow at a compound annual growth rate (CAGR) of 22.3%. It is estimated to be valued at about USD 229.8 billion by 2032, increasing from USD 24.6 billion in 2021 [21].

Our previous study proposed an initial framework for designing a BSN system [22,23]. In this paper, we focus on modeling the in-body sensor networks for Healthcare 4.0 by taking key parameters of BSNs into consideration. In particular, the process of body motion and blood circulation involved in measurement is formulated. Time-dependent parameters are included to represent changes of the related body part characteristics. The proposed model aims to utilize the minimum amount of chemical sensors and biosensors to collect sufficient measurements for healthcare purposes. Our previous study addressed hamstring injury detection [22,23]. As another technical enhancement, this work evaluates more medical cases to analyze the model’s performance. Typical injuries, such as tennis elbow, runner’s knee, shin splints, and heel inflammation, are investigated. More datapoints are provided in this work to analyze the performance of the enhanced BSN system model.

The rest of this paper is organized as follows: Section 2 overviews the BSN architecture that supports Healthcare 4.0. Its key elements are introduced one by one. Major functions of the key elements are reviewed. Section 3 presents the BSN model. It first explains the layers for biomedical information processing. Then, it focuses on formulating the goal, conditions, and constraints of BSNs using biomedical parameters. Factors in this model are elaborated to illustrate their impact on BSN system design. Section 4 investigates typical cases of human body injury detection. Multiple sets of simulations are conducted. Simulation results are analyzed and discussed. Our future research directions are discussed in Section 5. Conclusions of this work are drawn in Section 6.

## 2. Healthcare 4.0 and BSN Architecture

### 2.1. Healthcare 4.0 Elements

Healthcare systems are responsible for improving patient health and quality of care. Four vital functions of healthcare systems are provision of health care services, resource generation, financing, and stewardship [4,5]. Conventional healthcare practice deeply relies on human involvement. Typical activities are physical appointments, office visits, manual examinations, and on-site treatment procedures. Technological advances in medical devices, pharmaceutics, communications, networking, and other fields are driving the evolvement of healthcare practice. Healthcare systems are implicated in the rapid transition to an intelligent, scientific, and technological society. This society consists of a wide spectrum of business, industries, and governmental institutions. They conduct different activities to provide various healthcare services including, but not limited to, medication, caregiving, insurance, drug regulation, training, logistics, nutrition management, and safety management.

The latest effort is referred to as Healthcare 4.0. It is motivated by the successful progress of Industry 4.0. Key technologies from Industry 4.0, such as AI, big data, IoT, machine learning, and AR, are gradually introduced to the healthcare sector. Healthcare 4.0 is expected to synthesize all healthcare-related elements and transform healthcare with tech-driven applications and processes. It integrates various aspects of medical services and healthcare related industries with innovative technologies. The authors of reference [4] discussed a general platform for Healthcare 4.0. They focused on data security and patient privacy. A data storage and sharing method was proposed to conduct encryption and authentication from users’ smartphones. The authors of reference [5] researched homecare robotic system design for Healthcare 4.0. New features, such as cloud computing and motion capture, were investigated for potential homecare applications. Reference [7] enhanced platform security by using a method to protect highly sensitive data. In this manner, privacy breaches and cyberattacks from unauthorized users could be reduced. Reference [8] developed an integrated healthcare framework based on blockchain-based IoT technologies. The framework weights data properties to facilitate user data processing. The work in Reference [10] tackled the strategy of task distribution in the healthcare platform. It assigned multiarea tasks to different elements in the platform, improving efficiency and flexibility.

**Figure 1:**
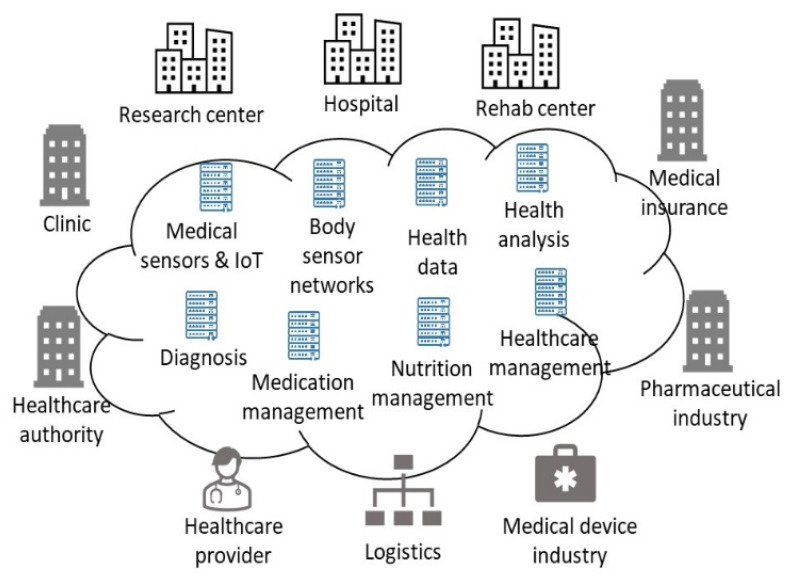
Key elements in Healthcare 4.0.

Our work aims to provide accurate data to realize Healthcare 4.0 for individual users. Figure 1 illustrates the key elements of Healthcare 4.0. Patients are the center of Healthcare 4.0. All services provided by Healthcare 4.0 share a common goal of patient care improvement. The stakeholder elements in Healthcare 4.0 include healthcare providers, authorities, and insurance companies. Hospitals, clinics, research centers, and rehab centers are the infrastructure elements. Pharmaceutical industry and device industry are the value chain elements which produce key supplies to healthcare services. Healthcare 4.0 enables the integration and interaction of the patients, stakeholder, infrastructure, and value chain. It changes the traditional business model into a patient-centric and data-driven intelligent society.

BSN systems provide patient data for Healthcare 4.0. They are the information source platform where original data are collected from the patients. In BSN systems, the chemical sensors and biosensors work together to monitor physiological statuses in various biological body fluids. These body fluids are interstitial fluid, blood, wound fluid, etc. Specific biological functions, such as respiration, blood pressure, temperature, heart rate, and electrocardiogram (ECG), are constantly measured by these sensors.

### 2.2. BSN Systems

**Figure 2:**
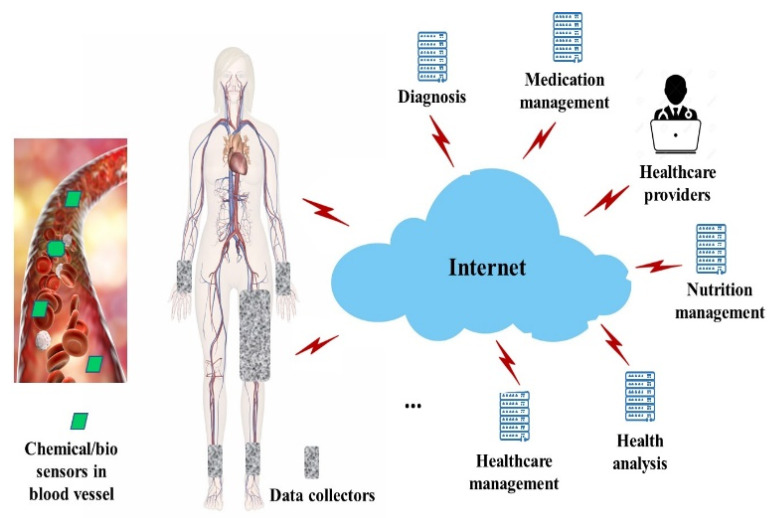
Body sensor network.

By analyzing and processing the data collected from these sensors, the BSN systems can radically increase the success rates of disease treatment and eventually improve the quality of life of patients. Figure 2 illustrates the BSN architecture. Chemical and biosensors enter the patient’s body via injection or drinking. Once injected or entering the blood circulation system, sensors flow with the blood through the patient’s body.

The in-body sensors collect data on respiratory rate, blood pressure, heartbeat rate, and body temperature. With technology advances in sensor design and material science, the latest generation of chemical sensors and biosensors can detect more bioinformation such as oxygen saturation, glucose levels, and cholesterol levels. All of these data are important bioparameters for healthcare.

From the viewpoint of healthcare, the in-body sensors provide insights into how patients’ bodies work. Healthcare providers and professionals can understand how medical conditions develop over time. Health problems are better managed at the early stage. Another benefit of the in-body sensors relies on the portability. The point of care is moved to wherever the patient is located. As real-time health information is collected and processed through the network, more responsive services can be provided. Bio data detected by sensors are collected via wearable devices placed around a few body places. In the example of Figure 2, wearable devices are placed around the patient’s hamstring, wrist, and ankle areas. These wearable devices are also called data collectors. Data collectors are designed with microcontrollers for data acquisition and transmission. They are smart electronic devices worn close on the surface of the patient’s skin.

Data detected by sensors are transmitted to data collectors when a sensor flows through an area covered by wearable devices. Collected data can be stored locally. They can also be transmitted to the Internet via communication interfaces in the data collectors. Other elements in Healthcare 4.0 use data provided by the BSN systems to conduct various tasks such as patient monitoring, diseases diagnosis, health analysis, nutrition management, as well as medication management.

## 3. BSN Modeling

Our model in this section is based upon the frameworks in IEEE 1906.1-2015 [24], IEEE 1906.1.1-2020 [25], and IEEE 8802.15.6-2017 [26]. This set of IEEE standards specify basic guidelines on sensor communications and performance evaluation. IEEE 1906.1-2015 specifies a conceptual framework on nanoscale communications. It supports information transmission for both waves and particles. The metrics are designed to evaluate communication performance. IEEE 1906.1.1-2020 creates a common data model using the next-generation language YANG. This model represents the fundamental physics of nano communication systems. It also defines data structure for communication control and management. IEEE 8802.15.6-2017 specifies the physical layer and necessary MAC of the wireless body area network. It defines the frequency bands, launch power, and data rate to support wireless communication within the surrounding area of the human body. We customize the conceptual framework and data model defined in these reference standards with a specific goal of in-body sensor network design and implementation. In particular, our model is derived from the BSN architecture proposed in Figure 2. We aim to use the minimum amount of sensor resources to achieve the required readouts for health monitoring and other purposes. The problem settings and constraints are developed with the biological and medical parameters applicable to the architecture in Section 2.

### 3.1. Information Processing in BSNs

**Figure 3:**
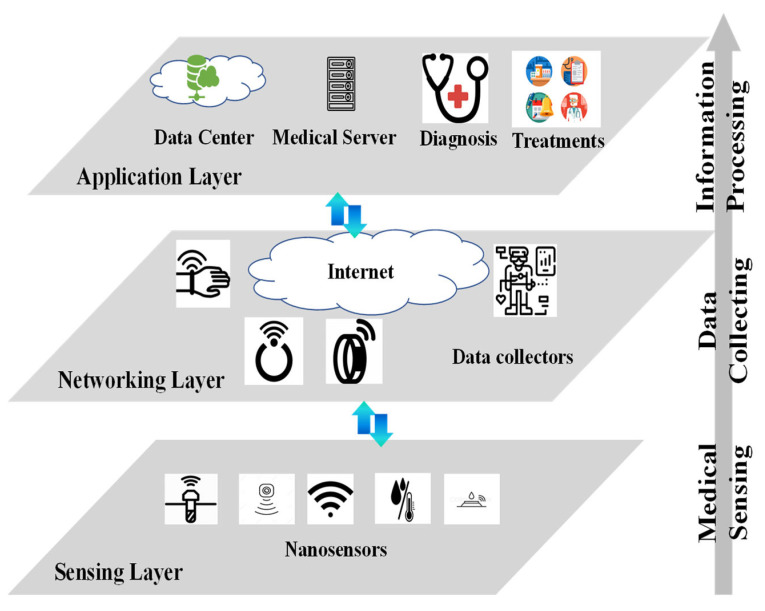
Major layers in BSN information processing.

In the proposed architecture shown in Figure 2, biomedical data are processed in multiple layers. Figure 3 shows the three layers of data processing: sensing layer, networking layer, and application layer.

The sensing layer lies at the bottom of the information processing chain. It consists of the sensors in the patient’s body. These sensors flow with the blood through the patient’s body. Depending on the actual implementation, the sensors could be in nanoscale or microscale. Figure 3 shows nano sensors as an example. These sensors detect physiological and medical information via physical, chemical, or biological methods. For example, to measure a particular compound’s concentration in the blood, the sensor sends a fluorescent signal that changes with compound concentration. The fluorescent signal can be detected through the skin. The received signal from the skin provides a way to measure and calculate the compound’s concentration. The detected information forms the raw data for healthcare services.

The middle layer is referred to as the networking layer. Its key elements are wearable devices, or data collectors. In this layer, wearable devices, such as watches, necklaces, wristbands, headbands, rings, and gloves, are deployed on the patient’s body to collect data from the in-body sensors. The wearable devices are designed with two types of communication interfaces. The southbound interface receives information from the in-body sensors, while the northbound interface transmits the healthcare information to the Internet. Note that the communication protocols implemented in the two interfaces could be different. This is because the signal coverage, transmitter power, receiver sensitivity, as well as data rate are different in the two interfaces. To continue the compound concentration detection example in the sensing layer, we assume a patient wears a smart wristband. The in-body sensor sends out fluorescent signal. The wristband keeps measuring the fluorescent signal through the skin. It maps the received fluorescent signal strength to a compound concentration value, and one readout is generated at the southbound interface. The readout could first be stored locally in the wristband memory. When protocol allows, the collected readouts would be transmitted to the Internet via the northbound interface. In some cases, the wearable devices can preprocess the collected data from in-body sensors, making edge computing or edge intelligence possible for the BSN system.

The top layer is the application layer. Unlike the sensing layer and the networking layer, the application layer provides more complicated functions of information processing. Healthcare data are stored with backups in the data center. The data center maintains healthcare service continuity when failures occur in the connections and even network nodes. In Healthcare 4.0, the data center also provides function modules to aggregate, reformat, update, and fuse the raw data. Medical servers use the data processed by the data center to help healthcare professionals with various tasks. Interrelated datasets that provide complementary views of the same phenomenon could be correlated to allow more accurate diagnosis. For example, physicians employ the tools installed in the medical servers to monitor patient health conditions, diagnose chronic injuries, and make treatment plans. Hospitals, clinics, and medical insurance companies employ the processed data to make strategic decisions on business operations.

### 3.2. BSN Model

The in-body chemical sensors and biosensors flow with the blood through the human circulatory system. Their velocity, *v* (cm/s), is the same as that of the human blood flow. It is modeled as:

*v*(*t*) = *Q*(*t*)/*A*(*t*), (1)

where *Q* is the blood volume flow per unit time expressed in units of volume per unit time (mL/s), and *A* is cross-sectional area of a blood vessel or a group of blood vessels expressed in units of square centimeter (cm^2^). Area *A* is calculated as *A* = *πr*^2^, where *r* is the radius of a single blood vessel or a group of blood vessels. Both *Q* and *A* are time-dependent because the vessel characters change when sensors flow through the circulation system.

The path length, *L*, of the circulation system is calculated as:

(2) $$L=\int_{0}^{T_{C}}v(t)dt$$

where *T_C_* is the time for blood to circulate through a human body. In each blood circulation circle, the chance that a sensor passes the wearable data collector area is:

*P_C_* = *w/L*, (3)

where *w* is the length of the wearable data collector.

A readout is collected when a sensor transmits its data while flowing with the blood through a data collector area. In this process, a transmission collision occurs if there are other sensors attempting to transmit data. The successful probability (i.e., *P_S_*) of collecting a readout from *n* sensors is expressed as:

*P_S_* = *P_C_* (1 − *P_C_*)*^n^*^−1^. (4)

During a healthcare monitoring time, *T_M_*, the successful number of readouts from *n* sensors is:

*S* = *n P_S_ T_M_*. (5)

Replacing *P_S_* using Equations (1)–(4), the successful readouts from *n* sensors in *T_M_* monitoring time are:

(6) $$S=nT_{M}\frac{w}{\int_{0}^{T_{C}}\frac{Q(t)}{\pi r^{2}(t)}dt}{\left(1-\frac{w}{\int_{0}^{T_{C}}\frac{Q(t)}{\pi r^{2}(t)}dt}\right)}^{n-1}$$

Chemical sensors and biosensors are a scarce medical resource. In-body sensor usage in patients should be limited to the extent of necessity. Each readout information transmission consumes energy from both the sensor and the data collector. Data transmission reduces the sensor lifetime. Our goal is to utilize the minimum number of sensors to collect sufficient readouts from the patient’s body.

Table 1 summarizes the key parameters used in the aforementioned BSN model.

In this model of in-body sensor networks, our goal is to minimize the value of *n* (i.e., sensor amount in the patient’s body) while achieving *S* successful readouts to satisfy *S* ≥ *S_min_*. *S_min_* is the minimum number of readouts required for a healthcare professional to make necessary decisions. The goal of the BSN modeling problem is stated in Table 2.

Parameters of our model can be divided into three categories. The first category is human body biological parameters. These include the blood circulation time (i.e., *T_C_*), the blood flow velocity (i.e., *v*(*t*)), the blood volume flow per unit time (i.e., *Q*(*t*)), the blood vessel cross-sectional area (i.e., *A*(*t*)), and the blood vessel radius (i.e., r(*t*)).

The second category represents the BSN system characteristics in Healthcare 4.0. It consists of the in-body sensor amount (i.e., *n*), the wearable data collector length (i.e., *w*), and the healthcare monitoring time (i.e., *T_M_*). The last category of parameters describes the BSN system performance. These parameters include the successful probability of collecting a readout (i.e., *P_S_*), the minimum readouts needed for healthcare (i.e., *S_min_*), and the number of successful readouts (i.e., *S*).

Various constraints should be taken into consideration when designing a BSN system. For example, there are minimum and maximum time limits for a sensor circulating the patient’s body (i.e., *T_C_*) [27,28]. Typical values are 45 s for the minimum and 60 s for the maximum [29,30]. Another constraint is that healthcare monitoring time should be much longer than the blood circulation time.

Other examples of BSN constraints are the blood vessel radius range of the patient’s body and the blood flow volume range [31]. As far as the blood vessel radius is concerned, it ranges from 4 μm in the capillaries to 12.5 mm for the aorta [32,33].

A heuristic algorithm named smart sensor selection (SSS) is proposed to search for the sensor amount *n* to satisfy the requirement of providing more than *S_min_* readouts. The pseudocode of SSS is shown in Algorithm 1. Note that the initial values of *P_C_*, *P_S_*, and *X* are all set as zero. SSS uses the monitoring time and the required readouts as input. It determines the number of sensors needed for BSN and generates readouts no less than the requirement. The SSS input is from the networking layer in Figure 3. The SSS output is the sensing layer formation in Figure 3. Therefore, the SSS algorithm is a tool to design the BSN system. Its complexity is mainly determined by *S_min_*, *T_M_*, *L*, *w*, and *v.*
**Algorithm 1:** Smart sensor selection    Input: *T_M_, S_min_*    Output: *n, S*    Initialize *P_C_*, *P_S_*, and *X* as zero    **for** *i* = 1: *T_C_* do    *r*(*i*) = random $\left(r_{min,}r_{max}\right)$    *Q*(*i*) = random $\left(Q_{min,}Q_{max}\right)$    *A*(*i*) = *πr*^2^(*i*)    *v*(*i*) *= Q*(*i*)/*A*(*i*)    *L = v*(*i* −1) + *v*(*i*)     **end**    Update probability *P_C_* that a sensor passes the wearable data collector area    **for** *m* = 1 do     Update successful probability *P_S_* of collecting a readout from *m* sensors     Update successfully collected readouts *X* from these sensors     **if** *X* ≥ *S_min_* // when readouts are no less than the requirement       Update *S*       Update *n*       Break    **end**    *m++* // Increase sensor amount when readouts are less than *S_min_*    **end**

## 4. Case Study and Performance Evaluation

Performance of the proposed model in Section 3 was evaluated in several cases. Simulations were conducted to study the BSN system performance. The parameters of each simulation set were configured based on the real cases from published references. They represent the common scenarios in which BSNs can be applied to help improve healthcare practice. The simulations are implemented on an Intel Core i7-1185G7 with MATLAB and Python 3.7.

### 4.1. Elbow Injury

The first set of simulations evaluated tennis elbow injury. This is a chronic body injury developing when tendons in the elbow are overloaded [34]. Due to repetitive wrist and arm motions, the forearm muscles and tendons become damaged from overuse. Tennis elbow injury often involves inflammation and/or micro-tearing of the tendons that join the forearm muscles on the outside of the elbow [35]. When muscles in the forearm are strained, tiny tears and inflammation can develop near the bony lump area of the elbow.

This chronic injury first leads to pain, burning, or an ache in the elbow. If the activity that causes the injury continues, the pain may spread to the wrist. The impacted elbow develops stiffness, and the range of elbow motion reduces. Tennis players are not the only people who suffer tennis elbow. It is common in other groups, such as plumbers, painters, carpenters, and butchers, whose jobs feature repetitive motions of the wrist and arm [36,37].

Typical parameters and constraint values of the tennis elbow injury were collected from references [34,35,36,37]. They were plugged into the model and algorithm in Section 3. Specifically, in our simulations on the tennis elbow injury, *T_C_* is set to 50 s, *v*(*t*) was set as a time-dependent random process in the range between 1.5 cm/s and 19 cm/s, and the data collector length *w* was assumed to be 20 cm to cover the elbow part. The monitoring time *T_M_* was configured as 10 min. We designed this set of simulations to evaluate the relationship between deployed sensors and collected readouts.

**Figure 4:**
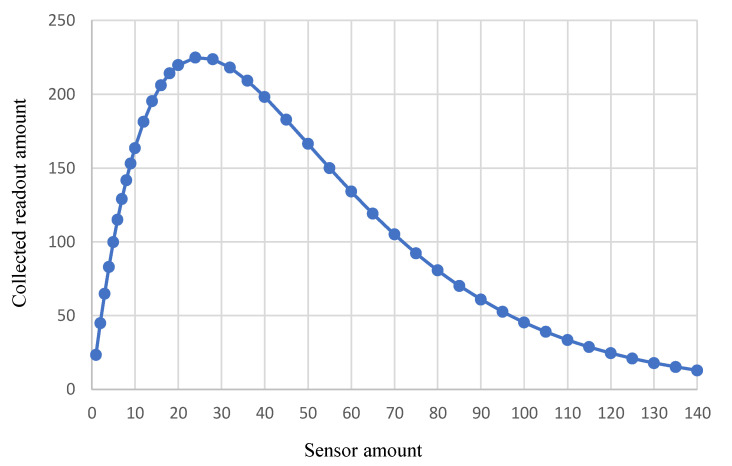
BSN performance for tennis elbow injury monitoring.

Figure 4 shows the collected readout amount changes as the in-body sensor amount varies. Assuming the minimum readouts needed to diagnose the tennis elbow injury, *S_min_*, is 100, in order to collect more than 100 readouts, the deployed sensor amount should be larger than 5 and less than 72. When 25 sensors are deployed in the patient’s body, a maximum readout amount of 225 is achieved within the 10 min monitoring time. After that, deploying more sensors would increase the chance of transmission collision, as more than one sensor could simultaneously transmit when flowing through the wearable data collector area. To meet the requirement of collecting 100 successful readouts, the most efficient design would be utilizing 6 in-body sensors to monitor bioparameters of the tendons and muscles in the elbow area. Note that in the elbow injury case simulated in Figure 4, the maximum successful readout is 225. Determinant factors of this maximum value include the blood circulation time and the healthcare monitoring time.

The first set of simulations illustrates the relationship between the BSN resource (e.g., sensor amount) and the performance (e.g., collected readouts). There are many factors, such as sensor transmission collision, blood circulation time, monitoring time, and blood velocity variation, that come into play in the BSN system performance. It is not a linear nor monotonic function between the sensor amount and the collected readouts. In other words, given a particular goal of healthcare, the deployed sensor amount should be carefully selected to meet the performance requirements.

The elbow injury case represents the BSN application in detecting a specific injury or disease. When a patient experiences typical symptoms that are associated with an injury or disease, healthcare professionals would use a wearable device to exam a particular part of the patient’s body. This helps to accurately diagnose the root reason before any treatment is selected.

### 4.2. Multiple Area Examination in a Single Routine

The second set of simulations are designed to evaluate multiple areas in a human body. This type of BSN application is valuable in health monitoring and multi-injury detection. Detecting injuries in runners may be used as an example. Runners often suffer from multiple common injuries. These injuries include runner’s knee, shin splints, and heel inflammation. Runner’s knee is caused by conditions that cause pain around the kneecap. Other than running, any activity that repeatedly stresses the knee joint can cause this disorder. This includes walking, skiing, biking, jumping, cycling, and playing soccer. Recent research shows that people who are overweight are also prone to this type of knee disorder [38,39]. Runner’s knee causes pain in and around the kneecap when patients are active, or after sitting for a long time with the knees bent [40,41,42]. Patients may hear rubbing, grinding, or clicking sound of the kneecap when bending and straightening knees.

Shin splints occur in athletes who have changed their training routines such as exercise duration, frequency, or intensity. The increased activity overworks the muscles, tendons, and bone tissue in the shin area [43]. The activity that causes shin splints is high impact and repetitive exercise of the lower legs [44]. Runners, dancers, and gymnasts often suffer from shin splints. People who have flat feet or high arches are at risk of shin splints [45,46,47]. Patients suffer with aching or dull pain down the front of one or both legs.

Causes of heel inflammation may include running, tightness in the calf area, Achilles tendonitis, shoes with poor support, sudden inward and/or outward turning of the heel, or landing on hard surfaces [48,49,50]. Other than exercise, certain medical conditions, such as obesity, an inactive lifestyle, and high or low arches of the foot, can also lead to heel inflammation [51,52,53].

In this set of simulations, our goal is to use one procedure to monitor the bioparameters of these three areas in the patient’s body using the same group of sensors. This would save the sensor usage as the same group of sensor resources are reused for the three areas. Furthermore, it reduces the monitoring time because only one course of action is needed to collect the required readouts. Typical parameters and constraint values of the injuries are collected from references [38,39,40,41,42,43,44,45,46,47,48,49,50,51,52,53]. Specifically, the blood circulation time, *T_C_,* is set to 60 s. The blood velocity, *v*(*t*), is set as a time-dependent random process in the range between 3 cm/s and 15 cm/s. The data collector length *w* is assumed to be 15 cm, 50 cm, and 10 cm to cover the knee, shin, and heel areas, respectively. The monitoring time *T_M_* is configured as 10 min.

**Figure 5:**
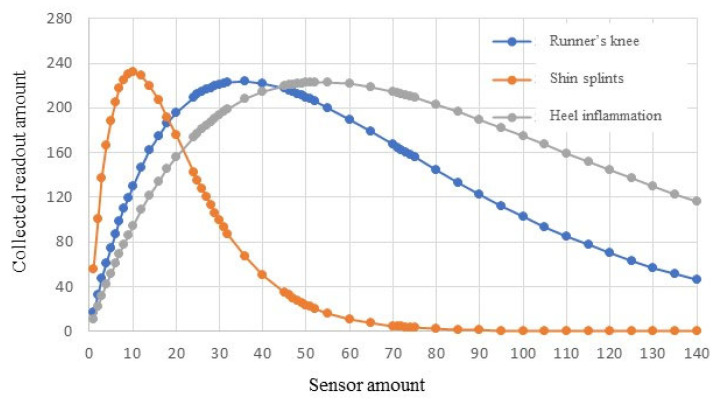
BSN performance for monitoring a group of injuries.

Simulation results are shown in Figure 5. In the case of runner’s knee, a maximum readout amount of 223 can be collected when using 36 sensors. The maximum readout amount of shin splints is 231 when 10 sensors are deployed. For heel inflammation, the maximum readout amount is 222 with 50 sensors in this set of simulations.

In order to collect 100 successful readouts from this patient, the runner’s knee case needs to use 8 to 100 sensors, the shin splint checkup requires 2 to 29 sensors, and the heel inflammation diagnosis needs at least 12 sensors. The most efficient design would be utilizing twelve in-body sensors to monitor bioparameters of the three areas in a patient’s body in one procedure.

This set of simulations verifies that the same group of sensors can be used in one procedure to fulfill multiple monitoring tasks for monitoring of the patient’s health or injury detection. In other words, related chronic injuries or diseases can be examined in a single procedure by sharing the same BSN resource. In the simulated cases, 3 parts (i.e., knee, shin, and heel) of the same patient are examined by using the same group of 12 sensors in a 10 min checkup procedure. The collected data provide 300 readouts (i.e., 100 readouts for the knee, 100 readouts for the shin, and 100 readouts for the heel). These readouts are sufficient for healthcare professionals to diagnose a patient’s condition. There is no need to conduct separate exams for these three injuries. Both the sensor resource and the exam time are saved.

The case shown in Figure 5 exemplifies the BSN application in physical examination to check a customer’s overall health. This type of wellness check is critical to identify any issues that may become medical concerns in the future, regardless of whether the customer is feeling symptoms or not. In the current physical exam routine, a series of tests need to be performed sequentially. In addition to visiting the primary care doctor’s office, a customer must make a lab appointments to collect blood and urine samples. In the near future, with Healthcare 4.0, multiple tests can be conducted simultaneously via BSNs to determine general health status. The exam results would be obtained more rapidly, e.g., in 10 min, in the case of Figure 5. Furthermore, the BSN system delivers much more readouts than the current physical exam, dramatically increasing the result accuracy.

### 4.3. Monitoring Time

The third set of simulations were designed to evaluate the impact of health monitoring time. In this study, the shin injury simulation settings were used with the goal of collecting at least 250 successful readouts. Typical parameters and constraint values of the shin injury simulations were collected from references [43,44,45,46,47]. Specifically, *T_C_* was set to 60 s. The blood velocity, *v*(*t*), was set as a time-dependent random process in the range between 3 cm/s and 15 cm/s. The data collector length *w* was assumed to be 50 cm to cover the shin area. In total, 3 different monitoring times, i.e., 10, 12, and 14 min, were configured to compare the successful readout amounts.

**Figure 6:**
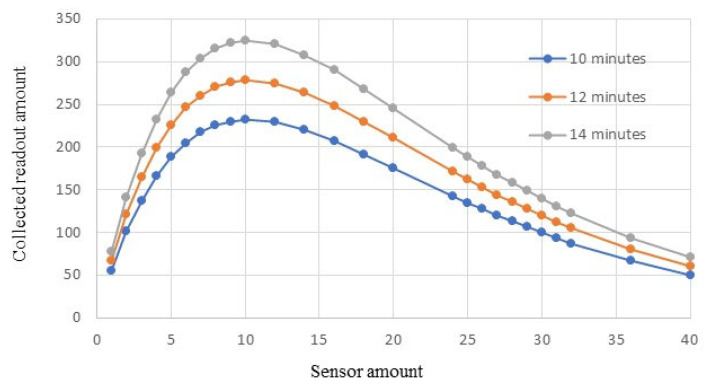
BSN performance in different monitoring times.

Simulation results are illustrated in Figure 6. When the monitoring time is set as 10 min, the performance is shown in the blue curve. The maximum number of readouts that can be successfully received during this monitoring time is about 231. It is impossible to meet the requirement of collecting 250 readouts or more from this procedure. In order to accumulate the required readouts, a longer monitoring time is necessary.

Figure 6 shows that when the monitoring time is increased to 12 min, the BSN system is able to collect 260 readouts by using 7 in-body sensors. This is illustrated in the orange curve in Figure 6. When the monitoring time is further increased to 14 min, the BSN performance is shown in the gray curve. The employed sensor amount can be reduced to 5, and the readout amount can still reach about 263.

This set of simulations demonstrates that the monitoring time is a determining factor of the maximum readouts generated by the BSN system. When designing the BSN system, it is critical to first decide the minimum monitoring time, making sure that the maximum readouts are larger than *S_min_*. After that, the step in which the number of sensors is determined can be conducted. Increasing the monitoring time will increase the maximum number of readouts.

## 5. Future Work

Our future work on this topic is planned in multiple fields.

First, more advanced technologies can be included in the architecture of our work. For example, BSNs collect a high volume of raw data from the sensors. Data fusion could be implemented in Healthcare 4.0 to integrate multiple data sources. This would generate more useful information and improve healthcare service reliability. In particular, potential data privacy and security concerns should be fully addressed. The BSN system is expected to ensure that collected user data is handled in accordance with applicable regulations and ethical principles. Towards this end, methods to protect BSNs from health data leakage and cyberattacks can be included to secure data transmission, storage, and processing.

Second, we would explore more medical cases for Healthcare 4.0. In addition to studying the injury cases, we can deep dive into a group of diseases. By taking advantages of in-body sensors, we plan to start with a blood-related disease, such as thalassemia or venous thromboembolism (VTE). The BSN architecture and system model need to be customized to describe the unique characteristics of the disease. BSNs can also be implemented in monitoring chronic conditions. This would effectively prevent diseases, such as heart attack, cancer, and diabetes, which are the leading causes of death and disability in our society.

Lastly, the BSN model needs enhancements. In this direction, our next step is to introduce more biomedical parameters for various physiological characteristics. Vitals, such as oxygen saturation, blood pressure, and glucose levels, can be derived or even formulated from new parameters in the model. Their biomedical constraints should be considered in designing the BSN system. In the current model, each data collector is assumed to possess one receiver to detect the sensor signal. This limit can be relaxed in our next step, as wearable devices are evolving with more smart functions and higher capabilities. These enhancements will increase the model complexity and, most likely, machine learning algorithms can be utilized.

## 6. Conclusions

This work applies the basic framework of sensor networks and communications specified in IEEE Standards 8802.15.6-2017 and 1906.1-2015 in the area of healthcare monitoring and examination. It employs in-body chemical and biosensors to collect medical data and physiological measurements from patients. An architecture of utilizing chemical sensors and biosensors for Healthcare 4.0 has been proposed. Key elements of the BSN system and their functions have been discussed.

Within the proposed architecture, information processing is conducted in the BSN system via a three-layer structure. A BSN model has been further developed to take the related bioparameters and BSN system performance into consideration. The goal is to design the BSN system for Healthcare 4.0 with minimum medical resources while delivering sufficient physiological measurement data. In this model, key parameters are formulated as time-dependent variables to represent in-body sensor movement. Biomedical and service constraints are analyzed.

Typical healthcare cases are investigated based upon the proposed model. Multiple sets of simulations have been conducted to evaluate the model’s performance. The first set of simulations illustrates the relationship between sensor amount and collected readouts. The second set of simulations reveals that a single group of sensors can be used for multiple tasks. The last set of simulations demonstrates the importance of selecting the monitoring time. Results have shown that the BSN system performance is decided by bioparameters of the patient as well as the BSN system healthcare parameters. The model in this work has provided feasible guidance on the BSN system design and healthcare data acquisition. Possible enhancements have been discussed as our future directions, in order to improve the BSN system design and performance.

## Figures and Tables

**Table 1:** BSN model parameters.

Item	Meaning and Unit
*n*	In-body sensor amount
*S*	Successfully collected readouts
*S_min_*	Minimum number of readouts for healthcare decisions
*v*	Sensor flowing velocity (cm/s)
*Q*	Blood volume flow per unit time (mL/s)
*A*	Cross-sectional area of a blood vessel (cm^2^)
*r*	Blood vessel radius (cm)
*T_C_*	Time for blood to circulate through a human body (s)
*T_M_*	Healthcare monitoring time (s)
*L*	Path length of the circulation system (cm)
*w*	Length of the wearable data collector (cm)
*P_C_*	Probability that a sensor passes the wearable data collector area
*P_S_*	Successful probability of collecting a readout from *n* sensors

**Table 2:** BSN modeling: problem goal and constraints.

Goal of BSN System Design	BSN System and Biomedical Constraints
Obtain the minimum *n* to satisfy *S* ≥ *S_min_*	*v*(*t*) = *Q*(*t*)/*A*(*t*)
	$L=\int_{0}^{T_{C}}v(t)dt$
	*P_C_* = *w*/*L*
	*P_S_ = P_C_*(1 − *P_C_)^n^*^−1^ $S=nT_{M}\frac{w}{\int_{0}^{T_{C}}\frac{Q(t)}{\pi r^{2}(t)}dt}{\left(1-\frac{w}{\int_{0}^{T_{C}}\frac{Q(t)}{\pi r^{2}(t)}dt}\right)}^{n-1}$ $T_{c}\in \left(t_{min,}t_{max}\right)$
	*T_M_ >> T_C_* $r\in \left(r_{min,}r_{max}\right)$ $Q\in \left(Q_{min,}Q_{max}\right)$

## Data Availability

Not applicable.
